# Non-Nutritive Sweeteners in the Packaged Food Supply—An Assessment across 4 Countries

**DOI:** 10.3390/nu10020257

**Published:** 2018-02-24

**Authors:** Elizabeth K. Dunford, Lindsey Smith Taillie, Donna R. Miles, Helen Eyles, Lizbeth Tolentino-Mayo, Shu Wen Ng

**Affiliations:** 1 The George Institute for Global Health, University of New South Wales, Sydney, NSW 2042, Australia; edunford@georgeinstitute.org.au; 2 Carolina Population Center, The University of North Carolina at Chapel Hill, Chapel Hill, NC 27516, USA; smithlp@email.unc.edu (L.S.T.); drmiles@email.unc.edu (D.R.M.); 3 Department of Nutrition, The University of North Carolina at Chapel Hill, Chapel Hill, NC 27516, USA; 4 National Institute for Health Innovation, School of Population Health, Tamaki Campus, The University of Auckland, Auckland 1072, New Zealand; h.eyles@auckland.ac.nz; 5 Nutrition and Health Research Center (CINyS), Instituto Nacional de Salud Pública (INSP), Universidad No. 655 Colonia Santa María Ahuacatitlán, 2100 Cuernavaca, Mexico; mltolentino@insp.mx

**Keywords:** non-nutritive sweeteners, sugars, processed foods, beverages, Australia, Mexico, New Zealand, United States

## Abstract

Increased interest among consumers in the reduction of dietary sugar intake has led to the wider availability of food products containing non-nutritive sweeteners (NNS). However, the extent to which NNS are currently being used by manufacturers to sweeten processed food and beverage products, and how NNS may be displacing added sugars as a sweetener is unknown. The current study utilized branded food composition databases from Australia, Mexico, New Zealand and the US to determine the percentage of processed food and beverage products for which there are nutrition data containing NNS and to compare total sugar density (g per 100 mL for beverages and g per 100 g for foods) between products with and without NNS. Ordinary least squares regression at the country-product level was performed to examine associations between presence of NNS and total sugar. Across all countries, 5% of products contained at least one NNS, with the highest prevalence among beverages (22%). Mexico had the highest percentage of products with NNS (11%), as compared to the United States (US) (4%), New Zealand (1%), and Australia (<1%). The presence of NNS was associated with lower mean total sugar density among beverages (range across countries: 7.5 to 8.7 g per 100 mL) and among foods (23.2 to 25.5 g per 100 g). Products with both added sugar ingredients and NNS had a lower overall mean total sugar density when compared to products containing only added sugar ingredients. Due to paucity of data on sales and market shares across these countries, our results do not reflect the extent to which consumers purchase NNS containing products. Continued monitoring of NNS in the food supply, extension of work from these data, and inclusion of market shares of products will be important as more countries introduce policies to reduce sugar.

## 1. Introduction

Over recent decades, the role of dietary sugar intake as a major driver of weight gain and type 2 diabetes has become more widely recognized among consumers, particularly regarding the consumption of sugar-sweetened beverages. Awareness is especially great in higher income countries where the intake of sugar-sweetened beverages and processed foods is prevalent, such as the United States (US), Australia, New Zealand, and Mexico [1,2,3,4,5]. Increased interest among consumers in the reduction of sugar intake has also led to wider availability of food products containing non-nutritive sweeteners (NNS; also termed artificial sweeteners, non-sugar sweeteners, and non-caloric sweeteners) [6]. In addition, obesity-prevention policies, such as sugary beverage taxes or front-of-package labels, may incentivise companies to replace added sugar ingredients with NNS [7]. 

NNS can be up to several thousand times sweeter than sucrose [8]; therefore, when compared with sugar, minimal amounts are needed to provide foods and beverages with a sweet taste. Although the intake of added sugars, and sugar-sweetened beverages in particular, is commonly associated with poor health outcomes [9,10], the association between NNS consumption and adverse health outcomes remains controversial [11,12]. Randomized controlled trials do not demonstrate an adverse relationship between NNS and energy intake or the increased consumption of sweet foods [13], yet several cohort studies, with varying degrees of potential for reverse causality, have linked NNS to increased body weight, type 2 diabetes, and other adverse cardio-metabolic outcomes [10,14].

In 2008, more than 30% of Americans reported consuming NNS on a daily basis, and this proportion is thought to be increasing [15]. Based on sales data, it is estimated that North America, Australia, and New Zealand consume double the amount of beverages containing NNS as compared to other regions, and the consumption of foods containing NNS alone or in combination with caloric sweeteners has increased dramatically over the past decade in the US alone [16,17,18]. A growing number of different types of NNS are available in the global food supply. Examples include aspartame, sucralose, and acesulfame K. However, to date, there have been no studies examining whether the proportion of packaged food and beverage products containing these ingredients differs between countries and regions around the world.

As consumers seek lower sugar and lower calorie food and beverage products, a better understanding of the types of foods that contain NNS ingredients is warranted. Further, an increasing number of products contain both added sugars and NNS [17]. A small number of previous studies have aimed to determine the extent to which NNS are being added to national food supplies [17,19]. However, it is not known how the use of NNS ingredients differs between countries, the extent to which NNS are currently being used by manufacturers to sweeten processed food and beverage products, and to what extent they may be displacing added sugars. Existing national food composition tables are limited in the information that they provide and do not generally provide insight into the types of NNS used by manufacturers during food processing. As such, the current study utilized branded food composition databases from four countries to determine the percentage of processed food products containing NNS and examined the relationship between the presence of NNS, added sugar ingredients, and total sugar density. This study’s data provides a first step towards monitoring changes in formulations over time and space, and future work linking these data to market share information can provide information on how sweeteners are shaping diets around the world.

## 2. Materials and Methods

Data for this study are from large branded food composition databases from Australia, Mexico, New Zealand, and the US that were available for research. For Australia, data from 2015 were derived from The George Institute’s FoodSwitch database, which contains branded food composition data for more than 20,000 packaged food items collected annually through in-store surveys [20]. For Mexico, branded food composition data collected in 2015–2016 by the National Institute of Public Health (INSP) were used. For New Zealand, data from 2016 were derived from The University of Auckland’s Nutritrack database, a brand-specific food composition database updated annually through in-store surveys. Finally, for the US, data spanning 2015–2017 were derived from Label Insight’s Open Data initiative. The initiative provides researchers with open access to granular food composition data not previously available to the research community. Researchers are granted the freedom to publish their findings based on Label Insight’s data without restriction. The database is updated daily and contains information on more than 290,000 barcoded food and beverage items (representing >85% of all products sold in the US food supply). Supplementary Table S1 describes the data collection protocols in each country in more detail.

### 2.1. Data Collection

Nutrient data were extracted for a total of 332,402 branded food and beverage items from among the four country databases. The following fields of information were extracted: Universal Product Code (UPC), brand name, serving size (g/mL), product description, full ingredients list, and total sugar density (g/100 g and/or g/serving).

### 2.2. Food Categorization

Foods were categorized into one of 12 major food categories that were largely based on the Global Food Monitoring Group’s categorization system [21], a global system that is used to examine the healthfulness of national food supplies. Baby foods and foods for specific dietary use (e.g., protein powders, nutritional supplements) were excluded from the current study since different regulations apply to these products as compared to other food categories. Supplementary Table S2 shows a description of each category included. Products within the ‘Dairy’ and ‘Beverages’ food categories were further broken down into subcategories to better differentiate between the types of food products containing NNS ingredients. Consequently, there were 24 separate food/beverage groups examined: ‘Beverage powder mixes’; ‘Coffee and tea’; ‘Cordials/syrups’; ‘Energy drinks’; ‘Fruit and vegetable juices’; ‘Milk drinks and milk substitutes’; ‘Soft drinks/sodas’; ‘Sports drinks’; ‘Waters’; ‘Yogurt and yogurt drinks’; ‘Bread and bakery products’; ‘Cereal and grain products’; ‘Confectionery’; ‘Convenience foods’; ‘Cheese’; ‘Cream’; ‘Dairy desserts’; ‘Ice cream and edible ices’; ‘Fruit, vegetables, nuts and legumes’; ‘Meat and meat alternatives’; ‘Sauces, dressings and condiments’; ‘Seafood and seafood products’; ‘Snack foods’, and; ‘Sugar, honey and related products’.

### 2.3. Determination of Presence of NNS and Added Sugar Ingredients

Products were classified as containing NNS and/or added sugar ingredients based on keyword searches within ingredient lists for each product. A detailed list of key terms is provided in Supplementary Table S3 and was compiled across the countries based on reviewing each country’s food labeling and additive regulations around sweeteners [22,23,24,25]. Briefly, NNS included artificial sweetener, aspartame, saccharin, sucralose, cyclamate, acesulfame K, stevia, and brand name versions of each sweetener. Added sugars included keywords, such as cane sugar, honey, agave, molasses, syrups, and fruit-juice concentrates (except when in 100% fruit juices). Following scientific nomenclature, sugar alcohols (e.g., erythritol) were considered neither NNS nor added sugars, but rather as another form of added sweetener and not included in the current study.

### 2.4. Statistical Analysis

The percentage of products from each country in each category containing NNS and added sugar ingredients was determined. Mean (SE) levels of total sugars per 100 g were calculated by category for each country. Differences in total sugar concentration between countries was examined using simple linear rank statistics, with a *p* value of <0.05 considered significant. The relationship between presence of any NNS (yes/no) and total sugar density (g per 100 g/mL) was examined using Pearson correlation coefficients.

To determine the degree of associations between presence of NNS and sugar density, ordinary least squares regression was performed at the country-product level separately for foods and beverages. Sugar density (g/100 g) was the outcome measure for two key model specifications. For Model 1, the key exposure measures were: the presence of NNS, country, and the interaction between presence of NNS and country, controlling for category. Results from Model 1 indicated to what extent NNS are displacing total sugars in products overall and if there were meaningful and statistical differences across countries for beverages and foods.

For Model 2, the presence of added sugar ingredients and its interaction with presence of NNS were included as key exposure measures. The coefficients from these additional variables indicated how total sugar density varied for products that contained both NNS and added sugar ingredients, contained only NNS or contained only added sugar ingredients relative to products that contained neither.

All statistical analyses were conducted using SAS version 9.4 (SAS Institute Inc., Cary, NC, USA). This secondary data analysis was deemed exempt from review by the University of North Carolina Institutional Review Board.

## 3. Results

Five percent (*N* = 14,865) of products from all four countries contained at least one NNS ingredient. Table 1 presents the breakdown of results by food category and country. Across all four countries, the highest prevalence of NNS was found in the ‘Beverages’ category, with ‘Energy drinks’, and ‘Beverage powder mixes’ containing the highest proportion within this category (56% and 50% respectively; Table 1). The ‘Dairy’ category contained the second highest proportion of products with NNS, with ‘Dairy desserts’ and ‘Ice cream and edible ices’ the predominant product types (6% and 5% respectively; Table 1). When examining results by country, Mexico had the highest proportion of total products with NNS (11%) compared to Australia (<1%), New Zealand (1%) and the US (4%; Table 1), and also the highest proportion in 15 of the 24 food/beverage categories that were examined. However, Mexico had the lowest proportion of products with NNS in three categories (‘Snack foods’, ‘Sports drinks’, and ‘Waters’) when compared to the other three countries.

Supplemental Table S4 presents the proportion of products by category and country containing at least one added sugar ingredient. Although the amount of added sugars is unavailable, the presence of added sugars is unsurprisingly found to be generally high across the countries, with some variability across countries by categories observed.

Mean total sugar density overall was lower in Australia (13.9 g/100 g) and New Zealand (13.8 g/100 g) as compared to Mexico (16.7 g/100 g) and the US (17.1 g/100 g), and was lower in these countries for 11 out of the 24 food categories (Table 2). Mexico had a higher mean total sugar density than the US in ‘Cereal and grain products’, ‘Sauces, dressings, and condiments’, and in three beverage subcategories (‘Soft drinks/sodas’, ‘Cordials/syrups’, and ‘Fruit and vegetable Juices’). The US in general, however, had the highest overall mean total sugar density, as well as the highest mean total sugar density across the majority of the food categories.

Table 3 shows the results from the two sets of models. Model 1 indicates that in the US, beverage products containing NNS had on average 8.7 fewer grams of sugar per 100 mL as compared to beverage products without NNS (*p* < 0.001). In addition, among NNS-containing beverage products, significant differences were found in the density of total sugar across countries. NNS-containing beverages in Mexico had 3.9 more grams of sugar per 100 mL than NNS-containing beverages in the US (*p* < 0.001), while Australia and New Zealand had similar sugar densities when compared to the US. However, beverages that did not contain NNS in Australia and New Zealand had significantly lower sugar density (3.4 to 4.2 fewer grams, both *p* < 0.001) as compared to the US. Among the food products that contained NNS, there were no statistically significant differences in total sugar density across countries. However, US food products containing NNS averaged 23.2 fewer grams of sugar (per 100 g) when compared to US food products without NNS (*p* < 0.001).

Model 2 results indicate that US beverage products containing NNS but not added sugar ingredients averaged 7.5 fewer grams of sugar (per 100 mL) as compared to US beverages that had neither NNS nor added sugar ingredients (*p* < 0.001). Conversely, beverages that contained added sugar ingredients as the only sweetener had 10.5 more grams of sugar (per 100 mL) when compared to beverages with neither NNS or added sugar ingredients (*p* < 0.001). Beverages sweetened only by NNS in Mexico had a higher overall sugar density than their counterparts in the US (difference: 4.8 g, *p* < 0.001). The sugar densities of beverages that contained both NNS and added sugar ingredients were non-significantly different from those that contained neither, suggesting that NNS may have become the main sweetener in beverage products containing both types of sweeteners.

Among food categories, the products that contained only added sugar ingredients had a higher overall total sugar density (+13.2 g, *p* < 0.001) than products containing neither NNS nor added sugar ingredients, while the sugar density from foods that contained both NNS and added sugars was at +10.3 g (*p* < 0.05) when compared to products with neither sweetener. This suggests some displacement of added sugars among food products.

Based on the prevalence of NNS across product categories and the available sample sizes, models were also examined for select categories: ‘Soft drinks/sodas’; ‘Fruit and vegetable juices’; ‘Waters’; ‘Yogurt and yogurt drinks’; ‘Dairy desserts’; ‘Ice cream and edible ices’, and; ‘Confectionery’. Table 4 presents the results from Model 1 (Panel 4a) and Model 2 (Panel 4b). As expected, for Model 1 across the seven categories, products in the US containing NNS as the only added sweetener had lower sugar density than products that do not contain NNS. Additionally, ‘Soft drinks/sodas’ that did not contain NNS were found in Australia and New Zealand to have 3 to 3.5 g per 100 mL lower sugar density than their US counterparts (*p* < 0.001). While Mexican ‘Soft drinks/sodas’ that did not contain NNS also had lower sugar density, this was a smaller difference (−0.5 g/100 mL, *p* < 0.05). Compared to US ‘Soft drinks/sodas’ that contain NNS, these beverages in Australia, Mexico and New Zealand had higher sugar densities, suggesting that US products may be more likely to contain combinations of NNS and added sugars.

Mexican ‘Fruit and vegetable juices’ and ‘Yogurt and yogurt drinks’ without NNS contained greater sugar densities than US products (+3.9 and 3.3 g/100 mL, respectively). However, Mexican ‘Fruit and vegetable juices’ and ‘Yogurt and yogurt drinks’ with NNS contained lower sugar densities than US products (−2.2 and −1.7 g/100 mL, respectively). ‘Waters’ without NNS in the US also contained more sugar per 100 mL than products in Australia, Mexico, and New Zealand. Also, when compared to US ‘Dairy desserts’ without NNS, products from Australia had −10 g/100 g less sugar.

For these select categories, Model 2 results were consistent with Model 1, with the exception that products in the US containing NNS as the only added sweetener were no longer significantly different than products that do not contain NNS for ‘Ice cream and edible ices’ and ‘Confectionery’. Meanwhile, ‘Soft drinks/sodas’, ‘Waters’, and ‘Yogurt and yogurt drinks’ that contained both NNS and added sugar ingredients had lower sugar densities (−1.5, −3.4 and −3.8 g/100 mL, respectively) than products that contained neither sweetener, suggesting that other sweeteners, such as sugar alcohols, may be included in these beverage types. However, sugar density for ‘Fruit and vegetable juices’ that contained both NNS and added sugars was 2.5 g/100 mL higher when compared to products with neither type of sweetener. This suggests some displacement of added sugars among ‘Fruit and vegetable juices’.

## 4. Discussion

The results presented in this paper highlight current use of NNS and added sugar ingredients in packaged food and beverage products from four countries over a similar time period (2015–2017). Monitoring the added sugar density and the types of NNS ingredients used in foods and beverages has been an ongoing challenge for health organizations around the world. Globally, limited research exists investigating the use of NNS ingredients in national food supplies [17,19]. This study found that, overall, 5% of products from Australia, Mexico, New Zealand, and the US contained NNS ingredients. 

Importantly, using a uniform definition for NNS given similar regulations on NNS use across the four countries, Mexico was found to have the highest proportion of products containing NNS ingredients overall (11%) as compared to Australia (<1%), New Zealand (1%), and the US (4%), and the highest proportion in 15 out of the 24 food and beverage categories examined. This finding is significant as previous research in this area has found that intake of NNS is highest in countries, such as the US, Australia, and New Zealand when compared to Latin American countries [18]. A reason for this inconsistency may be due to recent reformulation being undertaken by the food industry in Mexico in response to regulatory changes. For instance, in Mexico regulations were imposed in 2011 on foods and beverages allowed to be sold in schools, and a sugar-sweetened beverage and junk food tax was implemented in 2014 [26,27,28]. Due to these recent policy initiatives, it would be expected that the Mexican food and beverage industry may replace added sugar ingredients with NNS to avoid taxation and to be allowed to sell their products in schools. The US also has several state-based policies that have been introduced in recent years which could explain its higher use of NNS when compared to Australia and New Zealand, which do not yet have regulation related to the use of added sugars.

One other possibility for the US having a higher proportion of products with NNS compared to Australia and New Zealand is that the US has increased preference for “diet”, “low-calorie”, or “light” options in the wake of the obesity epidemic, with nearly half of the US overweight population reporting that they are on a diet [29]. Yet another possibility could be due to differences in sweetness preferences between the US and Australia/New Zealand. This possibility is supported by the fact that the US and Mexico in the present analysis, besides having larger proportion of products containing NNS, also had higher levels of total sugar concentrations (16.7 g/100 g and 17.1 g/100 g, respectively) than Australia (13.9 g/100 g) and New Zealand (13.8 g/100 g). It will therefore be important to monitor changes in total sugar concentration that may occur over time in Mexico and the US with the introduction of policies related to the use of added sugar.

Measurement difference might also account for differences found across countries, although it is unclear which approaches may prove to be more accurate or representative. Given that the New Zealand data collection protocol was based on the Australia data collection protocol, and the fact that these two countries have the combined food standards agency (Food Agency of Australia and New Zealand) that oversees the regulations on additives and sweeteners combined, it is not surprising that their results were very similar. The Mexico data sample was across more cities (as opposed to only one city each for Australia and New Zealand), and therefore may have a greater variety of products across regions. Finally, the US data were provided by a vendor that obtained their data directly from manufacturers and retailers and utilizes machine-learning technology to parse information from labels, rather than relying on manual data entry.

Significant variations in the proportion of products containing NNS were observed across different food categories. Specifically, ‘Dairy’ and ‘Beverage’ categories, such as ‘Energy drinks’, ‘Soft drinks/sodas’, and ‘Sports drinks’, had a relatively high proportion of products containing NNS. This finding is likely reflective of consumers seeking lower calorie/sugar-free product selections in these categories [30], as well as food technology constraints on which food categories the application of NNS work well in. Marketing of foods and beverages sweetened with NNS may also play an important role. A recent study found that in the US, 11% of beverages and 35% of foods contained claims of having low or no amounts of sugar, fat, sodium, or calories [31].

The present findings for Australia are consistent with previous research that showed less than 1% of products in a random sample of Australian foods contained NNS, the same as what was observed in the present analysis (<1%) [19]. Previous US research using data from 2005–2009, however, found a lower proportion of products containing NNS (1%) than the present analysis (5%) [16]. It is unknown whether this increase in NNS ingredient presence in the US is due to differences in the data used, or whether there has in fact been an increase in the use of NNS in the US since 2009. To our knowledge, there have been no previous studies examining the use of NNS ingredients in New Zealand or Mexico.

The presence of NNS was associated with lower mean total sugar density overall, which is to be expected as manufacturers increase the use of NNS ingredients to offset added sugar ingredients in their products [30]. Moreover, products with both added sugar ingredients and NNS also had a reduced overall mean total sugar density when compared to products containing only added sugar ingredients. This is an important result to consider in light of research showing that in some countries, like the US, the purchases of foods containing both added sugar ingredients and NNS, as well as products containing NNS alone, are increasing [17], meaning that at a population level it is likely total sugar intake from beverage sources could be decreasing [32]. Across the four countries in this analysis, beverage products that contained NNS averaged 8.7 fewer grams of sugar per 100 mL as compared to beverages that did not contain NNS, with beverage products containing NNS in Mexico having 3.9 more grams of sugar per 100 mL than the US beverages containing NNS. Meanwhile, beverages that did not contain NNS in Australia and New Zealand had significantly lower total sugar density when compared to their counterparts in the US.

The current study was limited to only four countries for which data were available for this research. Although results may not be globally representative, by including data from western countries, such as the US and Australia where research has shown that the sales of products containing NNS are high, as well as data from Mexico as a representative country for Latin America where sales of NNS have been shown to be lower than the US and Australia, a better understanding of the use of NNS in these countries is gained which has not previously been undertaken. Additionally, analyses at this time were unable to be weighted by market share across countries or within categories, thus limiting the ability to determine whether the higher proportion of products with NNS ingredients seen in Mexico and the US would remain after accounting for product sales [33]. As a potential estimate, earlier work in the US found that in 2013, 26% of beverages contained any NNS, and accounted for 27% of the beverage volume purchased by US households [18]. This is in comparison to the 22% of beverages found to contain any NNS in this study, which suggest that perhaps a similar proportion of NNS-containing beverages by volume are being purchased in the US. On the other hand, a study in New Zealand comparing sales weighted versus unweighted values for a range of nutrients, found that unweighted data overestimated exposure to total sugar (the study did not look at NNS) [33]. Finally, the analyses for this research relied on nutritional values reported on product labels that may not accurately represent what is in the foods.

Nonetheless, additional questions that can be answered with these data relate to how products containing various types of sweeteners relate to caloric changes, claims on products, and how these might vary across countries and categories. Future studies could also examine the prevalence of specific types of sweeteners across categories. Finally, it will be important to link these product-level data to data on household food purchases or individual intake to understand how changes in sweeteners in the food supply might affect diet and health.

## 5. Conclusions

This study found that on average in Australia, Mexico, New Zealand, and the US, NNS are present in 5% of products, but varies across countries. Mexico, which in recent years has enacted several policies designed to reduce intakes of calories and sugar, had the highest prevalence of products containing NNS. As expected, the higher presence of NNS was associated with lower mean total sugar density overall. However, the degree to which NNS displace added sugar differs both across countries and across foods vs beverages. There were interesting regional differences of note—Australia and New Zealand had a much lower presence of NNS and total sugar density across food and beverage categories; while Mexico had the highest presence of NNS and still had total sugar density values that were similar to the US, indicating regional difference in the sweetness of products available. This could be due to differences across countries in terms of taste preferences, consumer acceptance of different approaches towards sugar reduction, and/or legislation on the use of sweeteners, for example. Further monitoring of NNS and sugars in the food supply and what they represent in terms of overall sales across countries and within categories will be needed to understand the changes that may take place as the result of governmental actions, changing preferences, or other secular trends.

## Figures and Tables

**Table 1:** Sample size and percentage of products containing non-nutritive sweeteners (NNS) by country.

Food Category	Sample Size (*n*)					% Containing NNS				
	Australia	Mexico	NZ	US	Total	Australia	Mexico	NZ	US	Total
Beverages	1529	2957	1382	32,065	37,933	3.47	36.35	8.47	21.67	21.60
Beverage powder mixes	13	495	69	3669	4246	7.69	42.83	15.94	51.73	49.98
Coffee and tea	495	390	250	8929	10,064	1.21	11.03	2.80	2.40	2.68
Cordials/syrups	71	16	81	444	612	35.21	50.00	16.05	27.70	27.61
Energy drinks	43	13	76	436	568	23.26	38.46	25.00	64.91	55.81
Fruit and vegetable juices	462	582	364	7284	8692	1.30	18.56	6.87	14.42	13.68
Milk drinks and milk substitutes	313	510	313	5329	6465	0.32	16.27		7.49	7.47
Soft drinks/sodas	277	1342	379	6487	8485	1.81	50.82	5.28	29.23	30.68
Sports drinks	22	69	28	369	488		21.74	28.57	37.94	33.40
Waters	146	50	135	4447	4778		4.00	10.37	30.20	28.44
Yogurt and yogurt drinks	345	326	380	4272	5323		37.12	0.26	21.70	19.71
Bread and bakery products	1622	1705	1577	34,074	38,978	0.06	5.98		1.48	1.56
Cereal and grain products	1554	1991	1482	19,345	24,372	0.32	4.72	0.88	1.16	1.38
Confectionery	899	2017	873	23,180	26,969	2.56	14.77	2.06	6.10	6.50
Convenience foods	818	614	734	17,631	19,797		0.33	0.14	0.60	0.55
Dairy (non-beverage)	1674	2021	1989	32,244	37,928	0.06	11.42	0.6	5.77	5.53
Cheese	568	649	697	12,015	13,929		.		0.22	0.19
Cream	71	110	49	1035	1265		0.91		0.19	0.24
Dairy desserts	120	77	151	1741	2089		33.77		6.26	6.46
Ice cream and edible ices	257	349	399	7852	8857		4.87	2.76	5.04	4.79
Fruit, vegetables, nuts and legumes	2192	1575	1860	61,593	67,220		1.90	0.70	0.78	0.78
Meat and meat alternatives	631	817	1009	16,709	19,166		0.12		0.19	0.17
Sauces, dressings and condiments	1496	1731	1754	24,741	29,722	0.60	3.06	0.17	0.96	1.02
Seafood and seafood products	474	607	470	6163	7714		0.16		0.05	0.05
Snack foods	440	1698	498	13,267	15,903		0.24	1.00	1.41	1.23
Sugar, honey and related products	247	324	302	5827	6700	10.12	28.70	5.96	9.42	10.22
TOTAL	13,576	18,057	13,930	286,839	332,402	0.86	11.08	1.44	4.37	4.47

Notes: Product level nutrient data are from The George Institute’s FoodSwitch database (Australia 2015), the National Institute of Public Health (INSP) database (Mexico 2015–2016), The University of Auckland’s Nutritrack database (New Zealand 2016), and Label Insight’s Open Data initiative (United States 2015–2017).

**Table 2:** Mean (SE) total sugar density (g per 100 g or 100 mL) by food category and country.

Food Category	Mean (SE) Total Sugar Density (g per 100 g/mL)				
	Australia	Mexico	NZ	US	Total
Beverages	8.4(0.3)	13.4 (0.4)	8.6 (0.3)	12.5 (0.1)	12.2 (0.1)
Beverage powder mixes	21.0 (7.2)	44.5 (2.4)	5.6 (0.8)	38.8 (0.7)	38.6 (7.7)
Coffee and tea	10.6 (1.4)	14.1 (1.3)	13.5 (1.5)	12.0 (0.7)	12.4 (1.7)
Cordials/syrups	6.3 (0.9)	32.7 (9.8)	8.4 (1.3)	13.8 (0.8)	12.5 (0.8)
Energy drinks	7.8 (0.8)	7.2 (1.4)	8.2 (0.8)	6.8 (0.3)	7.1 (0.3)
Fruit and vegetable juices	9.2 (0.1)	12.4 (0.5)	9.2 (0.2)	9.3 (0.1)	9.5 (0.1)
Milk drinks and milk substitutes	6.6 (0.5)	14.7 (7.1)	6.5 (0.5)	12.5 (0.6)	12.0 (0.6)
Soft drinks/sodas	7.1 (0.3)	6.7 (0.2)	7.5 (0.2)	8.6 (0.1)	8.1 (0.1)
Sports drinks	6.4 (0.4)	11.2 (1.5)	3.8 (0.4)	4.4 (0.1)	5.5 (0.1)
Waters	3.3 (0.3)	0.4 (0.3)	2.7 (0.3)	3.5 (0.1)	3.4 (0.1)
Yogurt and yogurt drinks	10.8 (0.2)	13.4 (0.4)	8.8 (0.2)	10.2 (0.1)	10.3 (0.1)
Bread and bakery products	18.1 (0.4)	21.8 (1.6)	15.7 (0.4)	21.5 (0.1)	21.1 (0.1)
Cereal and grain products	9.0 (0.3)	15.9 (0.7)	9.9 (0.3)	11.7 (0.1)	11.8 (0.1)
Confectionery	44.9 (0.8)	48.0 (0.8)	46.9 (0.7)	56.5 (3.3)	55.1 (0.3)
Convenience foods	2.7 (0.1)	3.5 (0.2)	3.1 (0.1)	3.6 (0.1)	3.5 (0.1)
Dairy (non-beverage)	8.8 (0.2)	10.4 (1.9)	9.7 (0.2)	11.8 (0.8)	11.4 (0.1)
Cheese	1.7 (0.1)	1.4 (0.1)	1.5 (0.1)	2.0 (0.1)	2.0 (0.1)
Cream	4.0 (0.2)	8.2 (1.1)	4.1 (0.3)	5.4 (0.2)	5.5 (0.2)
Dairy desserts	16.4 (0.9)	17.1 (1.8)	25.0 (1.4)	25.1 (0.5)	24.3 (0.5)
Ice cream and edible ices	22.3 (0.3)	18.2 (0.3)	22.2 (0.3)	24.3 (3.0)	23.9 (0.0)
Fruit, vegetables, nuts and legumes	17.0 (0.5)	9.5 (0.4)	16.7 (0.5)	14.9 (0.2)	14.9 (0.2)
Meat and meat alternatives	1.9 (0.1)	0.9 (0.1)	1.9 (0.1)	2.7 (0.0)	2.5 (0.0)
Sauces, dressings and condiments	12.3 (0.4)	18.7 (1.6)	12.6 (0.3)	11.3 (0.1)	11.9 (0.1)
Seafood and seafood products	1.4 (0.1)	0.4 (0.1)	1.6 (0.1)	0.8 (0.0)	0.9 (0.0)
Snack foods	5.2 (0.4)	6.3 (0.3)	6.3 (0.5)	10.3 (0.2)	9.6 (0.2)
Sugar, honey and related products	69.5 (2.0)	49.1 (2.3)	64.3 (1.8)	63.1 (0.5)	62.7 (2.5)
TOTAL	13.9 (0.2)	16.7 (0.4)	13.8 (0.2)	17.1 (0.3)	16.8 (0.2)

Notes: Please refer to Table 1 for category and country-specific sample sizes.

**Table 3:** Coefficient estimates (SE) of total sugar density in beverages (g/100 mL) and foods (g/100 g).

Covariates	Model 1		Model 2	
	Beverages	Foods	Beverages	Foods
Australia: does not contain NNS ^1^	−3.36 *** (−3.36)	−2.18 (1.50)	−2.41 *** (0.67)	−2.00 (1.50)
Mexico: does not contain NNS ^1^	0.88 (0.88)	−0.89 (1.38)	2.07 ** (0.60)	0.13 (1.38)
New Zealand: does not contain NNS ^1^	−4.17 *** (0.66)	−1.96 (1.43)	−4.19 *** (0.65)	−1.85 (1.43)
US: Contains NNS (as only added sweetener) ^1^	−8.70 *** (0.39)	−23.24 *** (2.61)	−7.47 *** (0.59)	−25.48 *** (3.78)
Australia: Contains NNS ^2^	2.61 (3.85)	−10.43 (24.26)	1.24 (3.80)	−5.26 (24.26)
Mexico: Contains NNS ^2^	3.92 *** (1.07)	8.18 (7.45)	4.77 *** (1.06)	9.36 (7.46)
New Zealand: Contains NNS ^2^	2.00 (2.64)	4.16 (19.08)	1.60 (2.60)	7.33 (19.09)
Over 4 countries: Contains added sugar ingredients (as only added sweetener) ^3^			10.46 *** (0.36)	13.23 *** (0.74)
Over 4 countries: Contains NNS and added sugar ingredients ^3^			−0.57 (0.70)	10.30 * (4.85)
Total number of products	37,583	239,695	37,583	239,695

Notes: NNS = non-nutritive sweeteners. Beverage models control for beverage categories; foods models control for food categories. ^1^ Referent are US products that do not contain NNS. ^2^ Referent are US products that contain NNS. ^3^ Referent are products that contain neither added sugars or NNS ingredients. Model 1 examines the association between the presence of NNS with total sugar density among products between countries. Model 2 extends Model 1 with including the interaction of presence of NNS and the presence of added sugar ingredients across all four countries. *** *p* < 0.001; ** *p* < 0.01; * *p* < 0.05.

**Table 4:** Coefficient estimates (SE) of total sugar density for select food and beverage categories.

**4a. Model 2 Covariates**	**Soft Drinks/Soda ^4^**	**Fruit & Vegetable Juices ^4^**	**Waters ^4^**	**Yogurt & Yogurt Drinks ^4^**	**Dairy Desserts ^5^**	**Ice Cream & Edible Ice ^5^**	**Confect−Ionery ^5^**
Australia: does not contain NNS ^1^	−3.52 *** (0.33)	−0.62 * (0.28)	−1.26 * (0.61)	−0.30 (0.26)	−10.08 *** (1.72)	−2.78 (15.64)	−13.35 (15.47)
Mexico: does not contain NNS ^1^	−0.51 * (0.22)	3.93 *** (0.28)	−4.15 *** (0.82)	3.32 *** (0.34)	−	−6.34 (14.05)	−7.63 (11.30)
New Zealand: does not contain NNS ^1^	−3.00 *** (0.29)	−0.29 (0.32)	−1.51 * (0.59)	−2.36 *** (0.25)	−1.41 (1.55)	−2.54 (12.90)	−11.66 (15.40)
US: Contains NNS (as only added sweetener) ^1^	−8.00 *** (0.15)	−4.50 *** (0.20)	−2.95 *** (0.21)	−4.34 *** (0.17)	−20.71 *** (1.82)	−15.0 (12.88)	−49.77 *** (12.86)
Australia: Contains NNS ^2^	5.30 * (2.40)	−1.17 (2.37)	−	−	−	−	4.41 (93.74)
Mexico: Contains NNS ^2^	1.48 *** (4.44)	−2.15 ** (0.66)	2.57 (4.00)	1.70 ** (0.55)	−	5.06 (62.45)	12.47 (36.19)
New Zealand: Contains NNS ^2^	4.12 ** (1.23)	−1.31 (1.21)	0.79 (1.60)	6.97 (4.47)	−	3.24 (76.18)	3.32 (105.43)
*N*	7880	8273	3408	5048	1857	8453	24,158
**4b. Model 2 Covariates**	**Soft Drinks/Soda ^4^**	**Fruit & Vegetable Juices ^4^**	**Waters ^4^**	**Yogurt & Yogurt Drinks ^4^**	**Dairy Desserts ^5^**	**Ice Cream & Edible Ice ^5^**	**Confect−Ionery ^5^**
Australia: does not contain NNS ^1^	−2.62 *** (0.30)	−0.42 (0.28)	−1.17 * (0.51)	0.60 ** (0.22)	−9.47 *** (1.70)	−2.66 (15.65)	−9.34 (15.58)
Mexico: does not contain NNS ^1^	1.84 *** (0.22)	3.73 *** (0.28)	−1.28 (0.68)	3.48 *** (0.28)	−	−5.68 (14.15)	−5.77 (11.34)
New Zealand: does not contain NNS ^1^	−2.63 *** (0.27)	−0.35 (0.32)	−1.55 ** (0.49)	−1.50 *** (0.21)	−1.23 (1.53)	−2.34 (12.91)	−8.59 (15.47)
US: Contains NNS (as only added sweetener) ^1^	−3.80 *** (0.28)	−6.54 *** (0.31)	−1.27 *** (0.22)	−0.99 ** (0.35)	−7.89 * (3.99)	−8.15 (47.52)	−13.97 (26.02)
Australia: Contains NNS ^2^	1.38 (2.19)	1.23 (2.35)					2.24 (93.76)
Mexico: Contains NNS ^2^	−0.52 (0.31)	−1.63 * (0.66)	1.04 (3.33)	1.45 ** (0.46)		5.75 (63.30)	11.75 (36.22)
New Zealand: Contains NNS ^2^	1.01 (1.28)	0.40 (1.20)	1.62 (1.33)	5.62 (3.80)		4.45 (76.92)	6.04 (105.57)
Over 4 countries: Contains added sugar ingredients (as only added sweetener) ^3^	7.08 *** (0.24)	1.42 *** (0.14)	7.23 *** (0.20)	7.05 *** (0.16)	18.37 *** (3.06)	11.66 (28.89)	45.02 * (21.34)
Over 4 countries: Contains NNS and added sugar ingredients ^3^	−1.48 *** (0.32)	2.47 *** (0.38)	−3.35 *** (0.35)	−3.83 *** (0.37)	−8.78 (4.63)	−6.43 (48.77)	−18.56 (32.55)
*N*	7880	8273	3408	5408	1857	8453	24,158

Notes: NNS = non-nutritive sweetener. ^1^ Referent are US products that do not contain NNS. ^2^ Referent are US products that contain NNS. ^3^ Referent are products that contain neither added sugars or NNS ingredients. ^4^ sugar density measured as g per 100 mL. ^5^ sugar density measured as g per 100 g. Model 1 examines the association between the presence of NNS with total sugar density among products between countries. Model 2 extends Model 1 with including the interaction of presence of NNS and the presence of added sugar ingredients across all four countries. *** *p* < 0.001; ** *p* < 0.01; * *p* < 0.05.

## Supplementary Materials

The following are available online at http://www.mdpi.com/2072-6643/10/2/257/s1, Table S1: Detailed description of data collection for each country, Table S2: Food categorization system, Table S3: List of search terms used in analysis, Table S4: Percentage of products containing added sugar ingredients by country.
