# Supplementary material for: Non-Nutritive Sweeteners in the Packaged Food Supply—An Assessment across 4 Countries
Source: Nutrients. 2018 Feb 24;10(2):257. doi: 10.3390/nu10020257 (PMC5852833; doi:10.3390/nu10020257)
Supplement: Supplementary file 1 [file nutrients-10-00257-s001.docx]

**Supplementary Table S1:** Detailed description of data collection for each country.

| **Country** | **Data collection description** |
| --- | --- |
| **Australia** | The George Institute (TGI) FoodSwitch Branded Food Composition Database was utilised for analysis. This database holds extensive food product information for the Australian packaged food supply from 2011 to present. Each year the data are collected between August and December from the same four grocery retail stores (Coles, Woolworths, ALDI and IGA) in Sydney, Australia by a team of trained researchers. Data are collected using a smartphone application which links product barcodes with three photos of the product (front of pack, nutrition information panel (NIP) and ingredients list). Product information is then entered, reviewed and categorised within the database following an established quality assurance protocol.  Nutrient data were extracted from TGI Branded Food Composition database for 2015 for the current analysis. |
| **Mexico** | Information from 18,183 industrialized products available in the main urban cities of Mexico were obtained from November 2015 to May 2016. The following regions were included; northern part of the country: Baja California, Chihuahua, Saltillo, and Monterrey, and center region: Guadalajara, Cuernavaca and Mexico City.  The fieldworkers visited 147 stores, including hypermarkets, supermarkets, convenience stores and membership food stores. They took photographs of all available foods and beverages in the aisles. According to their characteristics, the products were classified into 25 food categories. Each product had taken 6 photographs of its package; 1) barcode, 2) front of the package, 3) GDA, 4) nutritional information (table format), 5) ingredients list and 6) price. The information contained in each photograph was captured in the RedCap programme, such online software contains a capture mask designed by the University of North Carolina staff and the information was placed in the server of the same institution. |
| **New Zealand** | The Nutritrack database holds labelling and composition information for items available in New Zealand supermarkets and is estimated to encompass approximately 75% of nationally available packaged food and non-alcoholic beverage products which display NIPs. Annual surveys are undertaken by trained fieldworkers to collect brand, barcode, nutrient, ingredient and front-of-pack labelling information for all packaged food and non-alcoholic products in four main supermarket stores in Auckland between February and April each year. The four store brands from which Nutritrack data are collected from represent the largest retail brands of the two main national supermarket retailers: Foodstuffs (54% grocery market share) and Progressive Enterprises (38% market share).  Data are collected directly from all packaged products displaying a NIP. Field workers use a custom-designed smartphone application (app) to scan product barcodes and photograph all surfaces of food packages. Photographs are used as source data to enter the following information into a secure, online database: Barcode, product name and brand, package size, recommended serve size, all mandatory values reported on the NIP, i.e., energy, protein, total fat, saturated fat, total carbohydrate, total sugars, and total sodium per 100 g or 100 mL (and other non-mandatory nutrient values, e.g., fibre, where reported on the NIP), ingredients, and front-of-pack labelling. Quality assurance procedures include: One in every 10 products entered into the database is randomly selected for a full quality assurance check against product photos; regular reports are run to identify extreme or missing values for major nutrients; and the categorisation of all products is checked to ensure consistency within and between years. Any identified errors are corrected. If the accuracy rate of key fields drops below 98.8% a further 10% of products is selected for quality checking until the accuracy rate is >98.8%. Data for 2016 were used for the present analysis. |
| **USA** | Data for the present study were derived from Label Insight (labelinsight.com), which is the largest publicly available branded food composition database in the USA. Label Insight launched the Open Data Initiative in 2017, which provides researchers with open access to granular food composition data. The database is updated daily and contains information on >200 000 barcoded food and beverage items (representing >80% of the US packaged food supply). Nutrient data were extracted for 295,606 barcoded food and beverage items from the Label Insight portal. The following fields of information were extracted: Universal Product Code (UPC), brand name, product description, serving size, energy content (calories/serve), total fat (g/serve), saturated fat (g/serve), total sugar (g/serve), sodium (mg/serve) and the full ingredient list.  Data were not included in analysis if they had missing or implausible nutrient values, were products that did not display a nutrition facts panel (NFP) (such as tea, coffee, fresh produce etc.) or were products that were considered to be variety packs with multiple NFPs. |

| Supplementary Table S2: **Food categorization system** | |  |
| --- | --- | --- |
| **Food group** | **Food category** | **Description** |
| **Beverages** | Milk drinks and milk substitutes | Flavored and unflavored dairy milk products |
|  |  | Flavored and unflavored soymilks |
|  |  | Flavored and unflavored oat, almond and other milks |
|  |  | Condensed, evaporated and powdered milk products (including coconut milk) |
|  | Yogurt and yogurt drinks | Fruit, flavored and natural yoghurts (full fat, reduced fat and skim varieties) including yoghurt drinks |
|  | Juices | Fresh and ambient fruit and vegetable juices |
|  | Soft drinks | Sugar-sweetened and artificially-sweetened soft drinks |
|  | Sports drinks  Energy drinks  Cordials/syrups | Sports electrolyte drinks  Energy drinks  Cordial and syrup beverage mixes |
|  | Waters | Plain and flavored waters |
|  | Coffee and tea | All coffee and tea products |
|  | Beverage powder mixes | All beverage mixes |
| **Bread and bakery products** | Bread | White, wholemeal and mixed grain/seed sliced bread and rolls |
|  |  | Fruit bread and fruit-based muffins/rolls |
|  |  | Wraps and other flatbread products |
|  |  | Turkish pide, bagels, English-style muffins, crumpets, pizza bases and other plain bread-based products |
|  | Biscuits and cookies | Filled and unfilled sweet biscuits |
|  |  | Flavored and plain crisp bread and crackers |
|  | Cakes, muffins and pastry | Scones, pikelets, doughnuts, cakes, sweet buns, pancakes, crepes, muffins (cake-style), slices etc |
|  |  | Cake, pikelet and pancake dry mixes |
|  |  | Sweet pastries (fresh, ambient, chilled and frozen) |
| **Cereal and grain products** | Breakfast cereal | Ready to eat breakfast cereals |
|  |  | Oats and other breakfast cereals that require heating |
|  |  | Other processed cereals (e.g. bran) |
|  | Cereal and nut-based bars | Plain, chocolate-topped and yoghurt-topped cereal-based bars |
|  | Noodles | Plain dry noodles |
|  |  | Savory/flavored dry noodle-based dishes |
|  | Pasta | Canned and ambient pasta and sauce (with and without meat) products (excludes frozen ready meals) |
|  |  | Packaged fresh pasta with sauce |
|  |  | Savory/flavored dry pasta-based side dishes |
|  |  | Plain dry pasta |
|  | Rice | Plain rice |
|  |  | Savory rice-based side dishes |
|  | Other breakfast products | All breakfast products not included in “Breakfast cereal” |
|  | Other cereal products | Flour and other unprocessed cereals (e.g. polenta, cous cous, bread crumbs, yeast) |
| **Confectionery** | Chocolate and sweets | Chocolate-based confectionery, sugar-based confectionery |
|  | Chewing gum | All sugar-sweetened and sugar-free chewing gums and bubble gum products |
| **Convenience foods** | Pizza | Frozen and refrigerated pre-packed pizzas |
|  | Soup | Canned, chilled and ambient soup products |
|  | Ready meals | Frozen, chilled and ambient pre-prepared meals |
|  | Meal kits | Kits with ingredients to put meals together |
|  | Pre-prepared salads and sandwiches | Chilled pre-prepared salads and sandwiches |
|  | Other convenience foods | Other pre-prepared foods not included in above categories |
| **Dairy** | Cheese | Feta, haloumi, parmesan and other high-salt cheeses |
|  |  | All types of full and reduced fat cheddar/Colby etc cheese including shredded, block or sliced |
|  |  | Soft cheeses such as cream cheese, ricotta and cottage cheese |
|  |  | Processed cheese slices and products |
|  | Cream | Thickened, sour and regular cream products |
|  | Dairy desserts | Dairy-based desserts (e.g. custards, rice puddings) |
|  |  | Dairy-based dessert mixes (e.g. powders) |
|  | Ice cream and edible ices | Dairy and non dairy-based ice cream varieties and edible ices |
| **Fruit, vegetables, nuts and legumes** | Fruit | Dried fruit products including coconut |
|  |  | Fruit-based bars |
|  |  | Fruit products canned in juice or syrup |
|  |  | Fruit gels, fruits in jelly and fruit puree |
|  | Jam and fruit spreads | Jams, marmalades and other preserves |
|  | Nuts and seeds | Salted and unsalted nuts and seeds |
|  | Vegetables | Canned tomato products |
|  |  | Canned beans and peas |
|  |  | Baked beans in tomato sauce (with and without additions) |
|  |  | Canned creamed, plain and sweet corn |
|  |  | All other canned vegetables |
|  |  | Pickled vegetable and olive products |
|  |  | Frozen potato-based products |
|  |  | Frozen vegetables |
| **Meat and meat alternatives** | Meat alternatives | Plain tofu and other meat-free alternatives |
|  |  | Meat-free products (e.g. meat-free sausages) |
|  | Processed meat | Pre-packed bacon products |
|  |  | Beef, pork, chicken and lamb sausages and chilled hot dogs |
|  |  | Pre-packaged sliced deli meats |
|  |  | Pre-packaged salami and cured meats |
|  |  | Beef, pork, chicken and lamb meat burgers |
|  |  | Canned meat products (excluding soup and pasta) |
|  |  | Frozen meat pies, sausage rolls and other meat-based pastry products |
| **Sauces, dressings and condiments** | Mayonnaise and salad dressings | Full and low-fat mayonnaise |
|  |  | Oil-based, vinegar-based and other types of salad dressing |
|  | Sauces | Table sauces such as tomato sauces and ketchups, sweet chilli, BBQ sauces |
|  |  | Steak, HP and Worcestershire sauces |
|  |  | Soy, fish, oyster and other Asian high-salt sauces |
|  |  | Mustard products |
|  |  | Marinade products |
|  |  | Meat accompaniments (e.g. apple, cranberry and mint sauces) |
|  |  | Plain and flavored tomato paste products |
|  |  | Asian and Indian flavored powdered, ambient and liquid meal-based sauces |
|  |  | Ambient and fresh pasta sauces |
|  |  | Recipe bases |
|  |  | Liquid and powdered gravies and stock |
|  | Spreads | Crunchy and smooth salted and unsalted peanut butter |
|  |  | Relishes, chutneys and pickles |
|  |  | Other savory spreads (e.g. vegetable spreads) |
|  |  | Pâté spreads |
|  |  | Sweet spreads |
|  |  | Yeast-extract spreads (e.g. vegemite) |
|  |  | Chilled and ambient dips and salsa |
| **Seafood and seafood products** | Canned seafood | All varieties of plain and flavored canned seafood |
|  | Chilled and frozen seafood | Chilled processed fish products (e.g. smoked salmon) |
|  |  | Coated frozen fish products (e.g. fish fingers) and uncoated fish products |
| **Sugar, honey and related products** | Honey and syrups | Honey, golden, maple and other syrups  Dessert toppings  Sugar and artificial sweeteners |
| **Snack foods** |  | Plain and flavored potato crisps |

**Supplementary Table S3:** List of search terms used in analysis

| **Non-nutritive Sweeteners** | **Added sugar** |
| --- | --- |
| acesulfame k | agave nectar/syrup/sap/juice |
| acesulfame potassium | beet sugar |
| advantame | brown rice syrup |
| alitame | brown sugar |
| altern | cane sugar/juice/syrup |
| aspartame | caster sugar |
| aspartame-acesulfame salt | castor sugar |
| brazzein | clintose |
| candy leaf | coffee crystal |
| curculin | coffee sugar |
| cweet | confectioners powdered sugar |
| cyclamate | confectioners sugar |
| cyclamic acid | corn sweet/sweetener/syrup/sugar syrup/glucose syrup |
| enliten | date sugar |
| equal | demerara |
| erylite stevia | dextrose |
| instasweet | dextrose monohydrate |
| kaltame | dextrose anhydrous |
| lumbah | d glucose |
| luo han guo | dried glucose syrup |
| luo han kuo | dried raisin sweetener |
| mabinlin | dri mol |
| monatin | dri sweet |
| monellin | edible lactose |
| monk fruit extract | flomalt |
| natra taste | fructose |
| necta sweet | fructose sweetener |
| neohesperidine dihydrochalcone | galactose |
| neotame | glaze and icing sugar |
| nutrasweet | glucose |
| osladin | glucose-fructose |
| oubli | glucose-fructose syrup |
| pentadin | glucose syrup |
| purevia | golden syrup |
| reb a | gomme |
| rebaudioside A | granular sweetener |
| rebiana | granulated sugar |
| saccharin | high fructose corn syrup |
| splenda | honey |
| stevia | honi bake |
| steviol glycoside | honi flake |
| sucralose | icing sugar |
| sugar leaf | invertase |
| sugar twin | invert(ed) sugar |
| sunett | isoglucose |
| sweetleaf | isomaltulose |
| sweet’n low | kona ame |
| sweet one | liquid sweetener |
| syclamate | loaf sugar |
| twin sweet | maltodextrin |
| truvia | maltose |
| thaumatin | malt sweetener/syrup |
| E 950 | maple |
| E 951 | maple sugar/syrup |
| E 952i | milk sugar |
| E 952 | mizu ame |
| E 954 | molasses |
| E 955 | nulomoline |
| E 956 | pancake syrup |
| E 957 | powdered dextrose |
| E 958 | powdered sugar |
| E 959 | raw sugar |
| E 960 | raw cane sugar |
| E 961 | rice sugar |
| E 962 | rice syrup |
| E 969 | saccharose |
| acesulfamo de potasio | soft brown sugar |
| acesulfame de potasico | soft white sugar |
| acido 3-amino-n-(alfa-carbometoxifenetil) succinamico | sorbose |
| acido ciclamico | sorghum |
| acido ciclamico (y sales de sodio potasio y calcio) | sorghum syrup |
| acido ciclohexilsulfamico | starch sweetener |
| acesulfamo k | sucanat |
| acesulfamo-k | sucrovert |
| acesulfamok | sugar beet |
| alitamo | sugar cube |
| aspartamo | sugar invert |
| ciclamatos de sodio | sugar white |
| ciclamato de calcio | sweet n neat |
| ciclamato de sodio | table sugar |
| de potasio y de calcio | treacle |
| glicosidos de estiviol | trehalose |
| ester 1-metilico de la n-l-alfa-aspartil-l-fenilalanina | trusweet |
| ester metil aspartilico de la fenilalanina | turbinado |
| ester metilico de aspartilfenilalanina | turbinado sugar |
| estevia | versatose |
| estevia rebaudiana | white sugar |
| esteviosido | xylose |
| esteviosidos de glicerol | d-xylose |
| extractos de estevia | azucar |
| neohesperidina dc | azucar blanco (granulado o refinado) |
| neohespiridina dihidrochalcona | azucar blanco suave |
| neotamo | azucar de cana crudo |
| rebaudiosido a stevioside | azucar de la leche |
| sacarina | azucar de mesa |
| sacarina de calcio | azucar en polvo |
| sacarina de potasio | azucar flor |
| sacarina de sodio | azucar glace |
| sacarina soluble | azucar invertido |
| sal de aspartamo y acesulfamo | azucar moreno |
| splenda | azucar moreno suave |
| sucralosa | azucar rubio |
| triclorogalactosacarosa | d glocosa |
|  | dextrosa |
|  | dextrosa monohidrato |
|  | dextrosa anhidra |
|  | dextrosa en polvo |
|  | fructosa |
|  | glucosa |
|  | glucosa-fructosa |
|  | isoglucosa |
|  | jarabe de arce |
|  | jarabe de glucosa |
|  | jarabe de glucosa-fructosa |
|  | jarabe de glucosa seca |
|  | jarabe de maiz |
|  | jarabe de maiz de alta fructosa |
|  | jarabes para hotcakes |
|  | lactosa |
|  | melaza |
|  | miel |
|  | nectar |
|  | sacarosa |
|  | sorbosa |
|  | xilosa |
|  | d-xilosa |

**Supplementary Table S4.** Percentage of products containing added sugar ingredients by country

|  |  | **% containing added sugar ingredients** | | | | |
| --- | --- | --- | --- | --- | --- | --- |
| **Food category** |  | **Australia** | **Mexico** | **NZ** | **US** | **Total** |
| **Beverages** |  | 36.49 | 30.03 | 59.48 | 30.16 | 31.48 |
| ***Beverage powder mixes*** |  | 84.62 | 39.80 | 71.01 | 34.21 | 35.61 |
| ***Coffee and tea*** |  | 21.82 | 27.95 | 65.20 | 4.82 | 8.05 |
| ***Cordials/syrups*** |  | 53.52 | 43.75 | 72.84 | 48.87 | 52.45 |
| ***Energy drinks*** |  | 23.26 | 38.46 | 50.00 | 21.56 | 25.88 |
| ***Fruit and vegetable juices*** |  | 24.46 | 27.49 | 37.36 | 31.45 | 31.06 |
| ***Milk drinks and milk substitutes*** |  | 41.85 | 26.27 | 43.13 | 44.32 | 42.72 |
| ***Soft drinks/sodas*** |  | 80.51 | 28.09 | 85.22 | 66.18 | 61.47 |
| ***Sports drinks*** |  | 90.91 | 46.38 | 53.57 | 57.18 | 56.97 |
| ***Waters*** |  | 23.97 | 2.00 | 28.89 | 19.81 | 20.01 |
| ***Yogurt and yogurt drinks*** |  | 71.59 | 52.45 | 70.79 | 66.41 | 66.20 |
| **Bread and bakery products** |  | 74.97 | 72.55 | 77.49 | 75.26 | 75.22 |
| **Cereal and grain products** |  | 45.50 | 39.03 | 43.52 | 43.54 | 43.29 |
| **Confectionery** |  | 83.87 | 70.45 | 87.29 | 78.99 | 78.78 |
| **Convenience foods** |  | 77.63 | 62.87 | 79.84 | 72.95 | 73.09 |
| **Dairy (non-beverage)** |  | 46.48 | 32.61 | 48.11 | 46.77 | 46.10 |
| ***Cheese*** |  | 4.93 | 2.62 | 5.16 | 13.23 | 12.00 |
| ***Cream*** |  | 16.90 | 24.55 | 18.37 | 17.58 | 18.18 |
| ***Dairy desserts*** |  | 90.83 | 62.34 | 89.40 | 77.66 | 78.70 |
| ***Ice cream and edible ices*** |  | 97.67 | 81.66 | 93.48 | 86.04 | 86.54 |
| **Fruit, vegetables, nuts and legumes** |  | 30.61 | 37.59 | 36.56 | 30.72 | 31.04 |
| **Meat and meat alternatives** |  | 78.29 | 45.78 | 71.85 | 65.77 | 65.65 |
| **Sauces, dressings and condiments** |  | 74.06 | 61.53 | 74.69 | 64.77 | 65.63 |
| **Seafood and seafood products** |  | 39.24 | 17.30 | 46.38 | 23.41 | 25.3 |
| **Snack foods** |  | 67.27 | 38.10 | 71.69 | 64.05 | 61.61 |
| **Sugar, honey and related products** |  | 78.95 | 47.84 | 83.77 | 81.76 | 80.10 |
| **TOTAL** |  | 55.97 | 46.13 | 61.28 | 52.51 | 52.67 |
